# Whey Protein Lipid Concentrate High in Milk Fat Globule Membrane Components Inhibit Porcine and Human Rotavirus *in vitro*

**DOI:** 10.3389/fped.2021.731005

**Published:** 2021-09-01

**Authors:** Marcia H. Monaco, Gabriele Gross, Sharon M. Donovan

**Affiliations:** ^1^Department of Food Science & Human Nutrition, University of Illinois, Urbana, IL, United States; ^2^Medical and Scientific Affairs, Reckitt Benckiser/Mead Johnson Nutrition Institute, Nijmegen, Netherlands

**Keywords:** milk fat globule membrane, rotavirus, whey protein lipid concentrate, anti-viral activity, infant formula

## Abstract

**Background:** The milk fat globule membrane (MFMG) is a complex milk component that has been shown to inhibit rotavirus (RV) binding to cell membranes *in vitro*. Herein, a whey protein lipid concentrate high in MFGM components (WPLC) and whey protein concentrate (WPC; control) were screened for anti-infective activity against porcine OSU and human Wa strains of RV in both the African Green Monkey kidney (MA104) and the human colorectal adenocarcinoma (Caco-2) cell lines.

**Materials and Methods:** Confluent cells were exposed to OSU or Wa RV in the presence of WPLC or WPC (control) at 0, 0.1, 0.5, 1.0, 2.5, or 5 mg/ml. Infectivity was detected by immunohistochemistry and expressed as % inhibition relative to 0 mg/ml. WPLC efficacy over WPC was expressed as fold-change. One-way ANOVA analyzed data for the independent and interactive effects of concentration, test material, and RV strain.

**Results:** Both WPLC and WPC exhibited concentration-dependent inhibition of human Wa and porcine OSU RV infectivity in MA104 and Caco-2 cells (*p* < 0.0001). WPLC was 1.5–4.8-fold more effective in reducing infectivity than WPC. WPLC efficacy was independent of RV strains, but varied between cell lines. WPLC and WPC at concentrations ≥0.5 mg/mL were most effective in reducing human Wa RV infectivity in MA104 cells (*p* < 0.0001).

**Conclusions:** WPLC decreased infectivity of two strains for RV which differ in their dependency on sialic acid for binding to cells. Inhibition was observed in the most commonly used cell type for RV infectivity assays (MA104) and an intestinal cell line (Caco-2). An effect on virus infectivity might be a potential mechanisms of action contributing to beneficial effects of supplementation of infant formula with MGFM reducing the risk of infections and consequently diarrhea incidence in infants.

## Introduction

Although rotavirus (RV) vaccination programs are now widespread, RV-associated diarrhea remains a leading cause of severe gastroenteritis and death among children aged <5 years (1, 2). RV vaccines are effective in preventing hospitalizations due to diarrhea, however, protection conferred by the vaccine differs across regions of the world with lower effectiveness in settings with high child mortality (3). Thus, investigation into non-vaccine methods for reducing the global burden of RV is warranted. Breastfeeding is associated with reduced incidence of diarrheal diseases in infants by supporting nutrition status and immune development (4, 5). In addition, specific human milk components may confer protection against infection by acting as decoy receptors to block the attachment of pathogens to host cell surfaces, including anti-RV secretory immunoglobulin A (sIgA) (6) and human milk oligosaccharides (7, 8). Over the past two decades, both *in vivo* and *in vitro* studies have demonstrated health benefits associated with Milk Fat Globule Membrane (MFGM) and its component proteins, carbohydrates, and fats (9, 10), including improved neurocognitive development, metabolism (11) and reduced infectious disease (12, 13). In addition, both the lipid and protein fractions of MFGM reduce RV infectivity *in vitro* (14, 15).

The MFGM surrounds the vesicle that envelops the fat droplet produced by the mammary gland, and is comprised of a monolayer of polar lipids (phospholipids and sphingolipids), interspersed proteins (many of them glycosylated), gangliosides, and cholesterol (16). MFGM composition maintains the lipid dispersion in the milk's aqueous phase and protects the globules from enzymatic degradation (16). Zanabria et al. (17) showed that the structural organization of MFGM plays an important role for its biological activity. The mass of the MFGM accounts for 2–6% of the total mass of the milk fat globule (18), with proteins accounting for 1% of the total milk fat globule mass (16). Proteomics analysis identified more than five hundred proteins (19), while more than 30 different phospholipid molecules were characterized in the polar lipid fraction of MFGM (16).

The composition of MFGM is dynamic and affected by lactation stage, maternal diet and genetics, gestational age, and environmental factors (20–22). In addition, MFGM composition varies across species (23). Proteomic analysis identified similarities and differences between human and bovine MFGM protein composition. For instance, lactadherin and butyrophilin are the most abundant proteins in the membrane of both species. Lactadherin in human milk was shown to be associated with reduced symptomatic RV infection in infants (24) and human, but not bovine, milk lactadherin reduced RV infectivity *in vitro* (15), suggesting a potential role of this MFGM component in the anti-RV activity of MFGM. In contrast, Hettinga and collaborators (25) identified that the human MFGM was more enriched with immunoglobulins than bovine MFGM, while bovine MFGM was more enriched with antibacterial proteins than human milk. Despite some differences between human and bovine MFGM composition, clinical studies have established benefits of bovine MFGM supplemented pediatric nutrition formulations on a wide range of outcomes in infants and children (9).

The objective of this study was to investigate the efficacy of whey protein lipid concentrate high in MFGM components (WPLC) to reduce the infectivity of two different strains of RV (human Wa and porcine OSU) using two well-established tissue culture models for RV infection, the monkey MA104 cell and the human Caco-2 cell lines. In addition, we compared the effectiveness of the WPLC against a standard whey protein concentrate (WPC) as a control.

## Materials and Methods

### Cell Lines

The African green monkey kidney (MA104) cell line (Cat# CRL-2378.1; American Type Culture Collection [ATCC], Manassas, VA) was generously provided by Dr. Mark Kuhlenschmidt (Department of Pathobiology, College of Veterinary Medicine, University of Illinois, Urbana). MA104 cells were grown in EMEM (Cat# 30-2003; ATCC) with 10% premium U.S.-sourced heat inactivated fetal bovine serum (FBS, Cat# 35-016-CV; Thermo Fisher Scientific, Hanover Park, IL) and 1% antibiotic-antimycotic (Cat# 15240; Thermo Fisher Scientific). The human colorectal adenocarcinoma cells (Caco-2 cells) were purchased from ATCC (Cat# HTB-37, RRID: CVCL_0025). Caco-2 cells were grown in EMEM as described above, 20% premium U.S.-sourced fetal bovine serum (Cat# 35-015-CV; Thermo Fisher Scientific) and 1% Penicillin-Streptomycin (Cat# 17-602E; Lonza, Walkersville, MD, USA). After infection with RV, Caco-2 cells were maintained in the media as described above with the addition of 10% FBS.

### Rotavirus Strains

A sialidase-sensitive group A porcine RV OSU strain (P9[7], G5) was propagated in young pigs and purified by sucrose gradient centrifugation as described in Rolsma et al. (26). The sialidase-insensitive group A human RV strain (Wa) (tissue culture adapted, VP7 serotype 1 [G1]) was purchased from ATCC. The RV suspensions were treated with trypsin at a final concentration of 10 μg/ mL (Sigma Chemical Co., St Louis, MO, USA) for 30 or 60 min at 37 °C to activate the OSU or Wa virus, respectively, prior to cell infection.

### Whey-Protein Lipid Concentrate (WPLC) and Whey Protein Concentrate (WPC)

Multiple separately manufactured batches of a WPLC-MFGM-10 (Lacprodan; Arla Foods Ingredients, Viby J, Denmark) and a whey protein concentrate devoid of MFGM (WPC-80; Leprino Foods, Denver, CO, USA) were provided by Mead Johnson Nutrition/Reckitt (Nijmegen, The Netherlands). Stock solutions (10 mg/mL) of each material were prepared with serum-free EMEM and 1% antibiotic solution. The protein content (%wt/wt basis) of the WPLC and WPC preparations are shown in Supplemental Table 1.

### *In vitro* Rotavirus Infectivity Assay

The *in vitro* assay was a modified protocol as described in Hester et al. (7). MA104 cells were seeded in fibronectin-coated 24-well plates at 50 x 10^3^ per well. Media was changed every other day until cells reached confluence (4 days). Caco-2 cells were seeded in 24-well plates at 25 x 10^3^ per well. Confluence was reached within 5 days, and cells were allowed to differentiate for seven additional days before RV infection. The trypsin-treated porcine OSU RV (0.75 x 10^3^ focus forming units [FFU]) and human RV Wa (35 x 10^3^ FFU) were mixed with WPLC or WPC at 0.1, 0.5, 1.0, 2.5, and 5.0 mg/mL final concentration for 20 min before the application to the confluent cells. Before the infection, MA104 and Caco-2 cells were rinsed twice with serum-free EMEM, and then 100 μL/well of WPLC/RV or WPC/RV mixtures was dispensed to wells in quadruplicates. After 60 min, the WPLC or WPC/RV mixture was removed and replaced with EMEM plus 10% FBS and plates were incubated for 24 h at 37°C in a 5 % CO_2_ incubator. Experiments were performed in triplicates for each batch of WPLC and WPC at separate times.

Quantification of the RV-infected cells was performed by peroxidase immunocytochemical detection of virus progeny (27). Cells were fixed in methanol: acetic acid (9:1) and then washed with 70% followed by 50% ethanol solution for 5 min each. Endogenous peroxidase was blocked with 3% H_2_O_2_ followed by 5% normal horse serum (Vector Laboratories Inc., Burlingame, CA). The cells were incubated with a primary goat polyclonal anti-RV (Abcam Cat# ab20036, RRID:AB_777748) at 1:500 dilution overnight at 4°C. A biotinylated horse anti-goat IgG diluted at 1:200 was used as a secondary antibody (Vector Laboratories Cat# BA-9500, RRID:AB_2336123). Positive cells were detected with universal VECTASTAIN® Elite ABC-HRP Kit and DAB (3,3'-diaminobenzidine) Substrate kit (Vector Laboratories). Stained cell images were captured in Zeiss AxioZoom V16 (Carl Zeiss Microscopy, LLC, White Plains, NY, USA) at resolution of 32 X (7.20 mm^2^ area) and 63 X (2.91 mm^2^ area) for MA104 and Caco-2 cells, respectively. Viral infectivity was calculated by dividing the number of FFU (obtained with AxioVision 4.8, Carl Zeiss), at each WPLC or WPC concentration by the number of FFU at 0 mg/ml (% infectivity). Fold-change reduction of infectivity due to the MFGM in WPLC was calculated by dividing the average WPC % infectivity by WPLC % infectivity at each concentration tested.

### Statistical Analysis

Data were analyzed by one-way ANOVA via the MIXED procedure in SAS 9.4 (SAS Institute, Cary, NC) with Tukey-Kramer adjustments for the independent and interactive effects of concentration and test material. Data were tested for normality (UNIVARIATE procedures in SAS) and square root transformation was applied in the absence of data normal distribution. Data are expressed as means ± SEM and significance was set to *p* < 0.05, while a significant trend was considered when 0.05 > p < 0.1.

## Results

### MA104 Cell Experiments

Three batches each of WPLC and WPC were tested for differences in their ability to affect viral infectivity using MA104 cells and porcine RV OSU (Supplemental Figure 1). Two out of the three WPLC or WPC preparations did not differ in their ability to reduce RV infectivity. Thus, to limit the number of samples, from these three batches WPLC batches 1 and 3 and WPC batch 1 were selected for the remaining of the experiments. We confirmed that WPLC batches 1 and 3 did not differ in their ability to reduce infectivity when tested in Caco-2 cells with porcine or human RV, or MA104 cells with human RV (data not shown). Therefore, WPLC data throughout the experiments represent the mean of batches 1 and 3.

**Figure 1:**
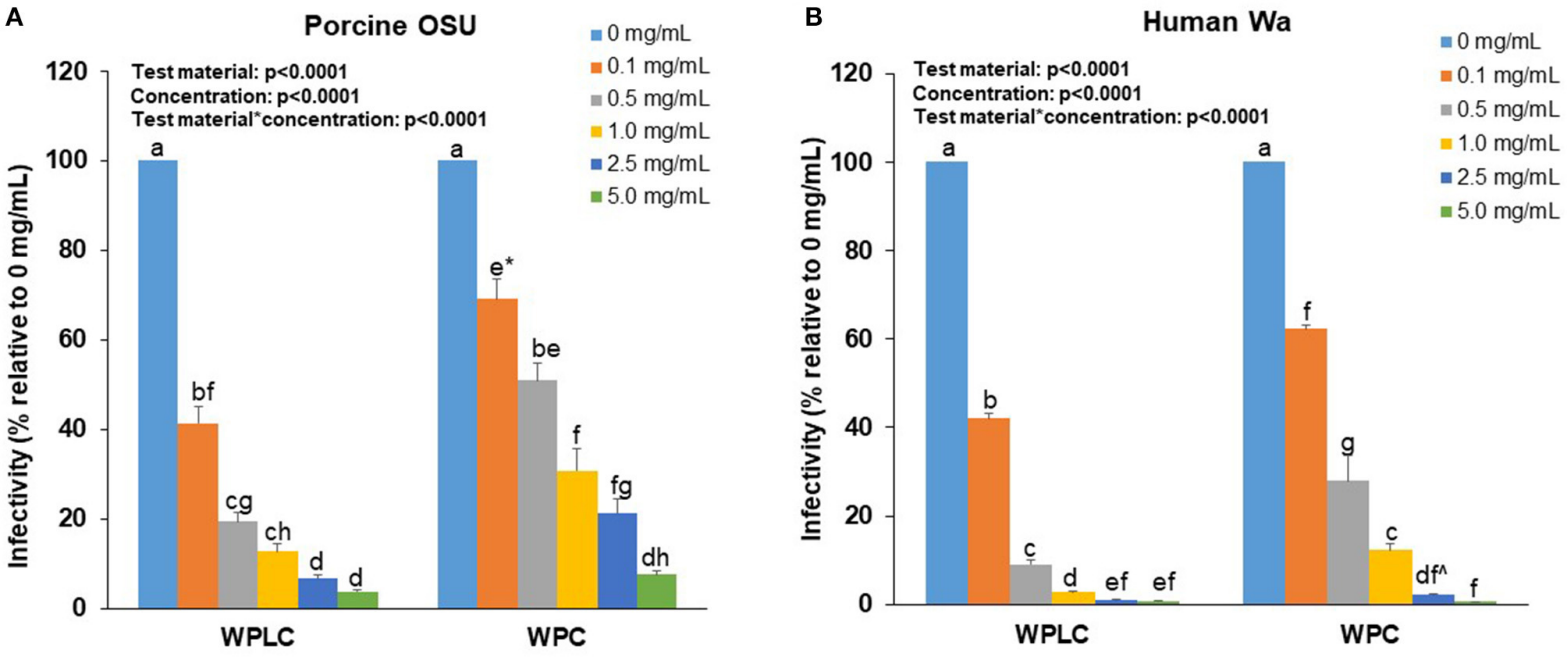
Concentration-dependent reduction of porcine OSU **(A)** or human Wa **(B)** RV strains infectivity in MA-104 cells by WPLC and WPC. Data are represented as means ± SEM of three separate experiments. Infectivity was calculated as % of FFU at each concentration relative to FFU counted in cells infected with RV alone (0 mg/mL). Statistical analysis assessed test material, concentration and test material^*^concentration effects. Unlike letters represent significant differences (*p* < 0.05) between concentrations within a test material and between WPLC and WPC. ^*^ Difference between WPC 0.1 and 0.5 mg/mL is at trend level (*p* = 0.058). ^∧^Difference between WPC 2.5 and 5.0 mg/mL is at trend level (*p* = 0.067). RV, rotavirus; WPC, whey protein concentrate; WPLC, whey protein lipid concentrate.

A concentration-dependent reduction in RV-infectivity was demonstrated with the human and porcine RV strains in MA104 cells by exposure to 0.1, 0.5, 1.0, 2.5, and 5.0 mg/mL of WPLC or WPC (Table 1). Both WPLC and WPC reduced % infectivity in a concentration-dependent fashion regardless of the strain used. The infectivity rates of porcine OSU RV in the presence of WPLC ranged from 41 to 3.8% and from 69 to 7.5% with WPC. A greater reduction in infectivity was observed when cells were incubated with human Wa RV plus WPLC or WPC, especially at concentrations ≥0.5 mg/mL, ranging from 42 to 0.7% to 62 to 0.6%, respectively. In addition to a concentration effect, there was a significant independent test material effect and significant interactive effect between concentration and test material (WPLC and WPC), regardless of the RV strain (Figure 1). WPLC was more efficacious in reducing porcine OSU infectivity than WPC at the concentrations below 5.0 mg/mL. However, when cells were infected with human Wa strain RV, the % infectivity between the two materials differed at the 0.1, 0.5, and 1.0 mg/mL concentrations.

**Table 1:** Porcine RV OSU and Human RV Wa Infectivity (%) in MA104 Cells modulated by co-incubation with either WPLC or WPC.

**RV Strain**	**Test material**	**WPLC or WPC Concentration (mg/mL)**					
		**0**	**0.1**	**0.5**	**1.0**	**2.5**	**5.0**
Porcine OSU	WPLC	100^a^	41.3 ± 3.7^b^	19.4 ± 1.9^c^	12.7 ± 1.8^c*^	6.6 ± 0.9^d^	3.8 ± 0.3^d^
	WPC	100^a^	69.1 ± 4.6^b^	50.9 ± 5.3^c^	30.8 ± 4.8^d^	21.2 ± 3.4^d^	7.5 ± 6.2^e^
Human Wa	WPLC	100^a^	42.1 ± 1.5^b^	9.1 ± 1.0^c^	2.7 ± 0.3^d^	0.9 ± 0.1^e^	0.7 ± 0.2^e^
	WPC	100^a^	62.3 ± 3.7^b^	27.9 ± 0.4^c^	12.3 ± 1.5^d^	2.2 ± 0.2^e^	0.6 ± 0.05^f^

*Data is expressed as means ± SEM. Values are % infectivity relative to 0 mg/mL*.

*Unlike letters indicate differences between the concentrations within each preparation at p < 0.05*.

* *Indicate statistically significant trend (0.5>p < 0.1) between WPLC, 0.5 and 1.0 mg/mL*.

**Figure 2:**
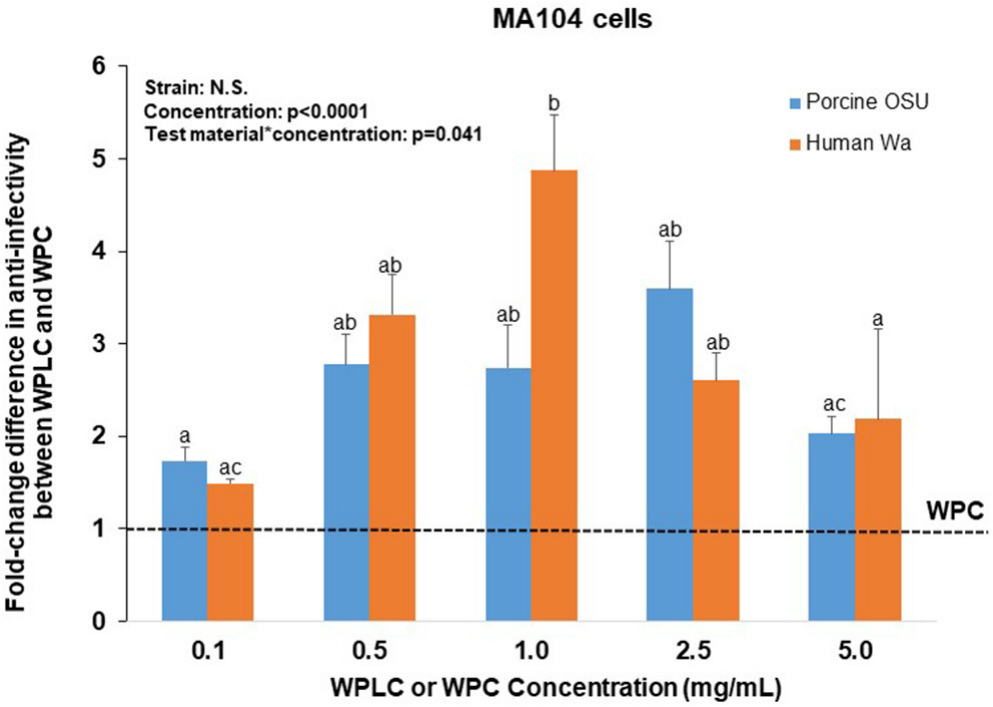
Fold-change difference in anti-RV activity of WPLC and WPC when MA104 cells were infected with porcine RV OSU or human RV Wa. Fold-change represents the ratio between WPC and WPLC efficacy to reduce % infectivity at each concentration tested. Data are represented as means ± SEM of three separate experiments. Unlike letters represent significant differences (*p* < 0.05) among concentrations. The largest difference in efficacy of WPLC over WPC to reduce infectivity of OSU and Wa strains was found at concentrations 1.0 and 2.5 mg/ml in MA104 cells. RV, rotavirus; WPC, whey protein concentrate; WPLC, whey protein lipid concentrate.

To further assess the efficacy of WPLC over WPC, the fold-change difference was calculated for both RV strains (Figure 2). The ability of WPLC to reduce infectivity relative to WPC was concentration-dependent and ranged from 1.5 to 4.9-fold. The largest difference in efficacy between WPLC and WPC, demonstrated by the highest fold-change difference, was at inhibiting human Wa and porcine OSU RV at 1.0 and 2.5 mg/mL, respectively. Fold-change did not differ among the doses when cells were infected with porcine OSU. In the presence of human Wa, WPLC at 1.0 mg/mL differed significantly from 0.1 to 5.0 mg/mL, but not the 0.5 and 2.5 mg/mL concentrations. WPLC was more effective than WPC in reducing RV infectivity under all conditions tested (i.e., fold-change difference >1 in all cases).

### Caco-2 Cell Experiments

**Figure 3:**
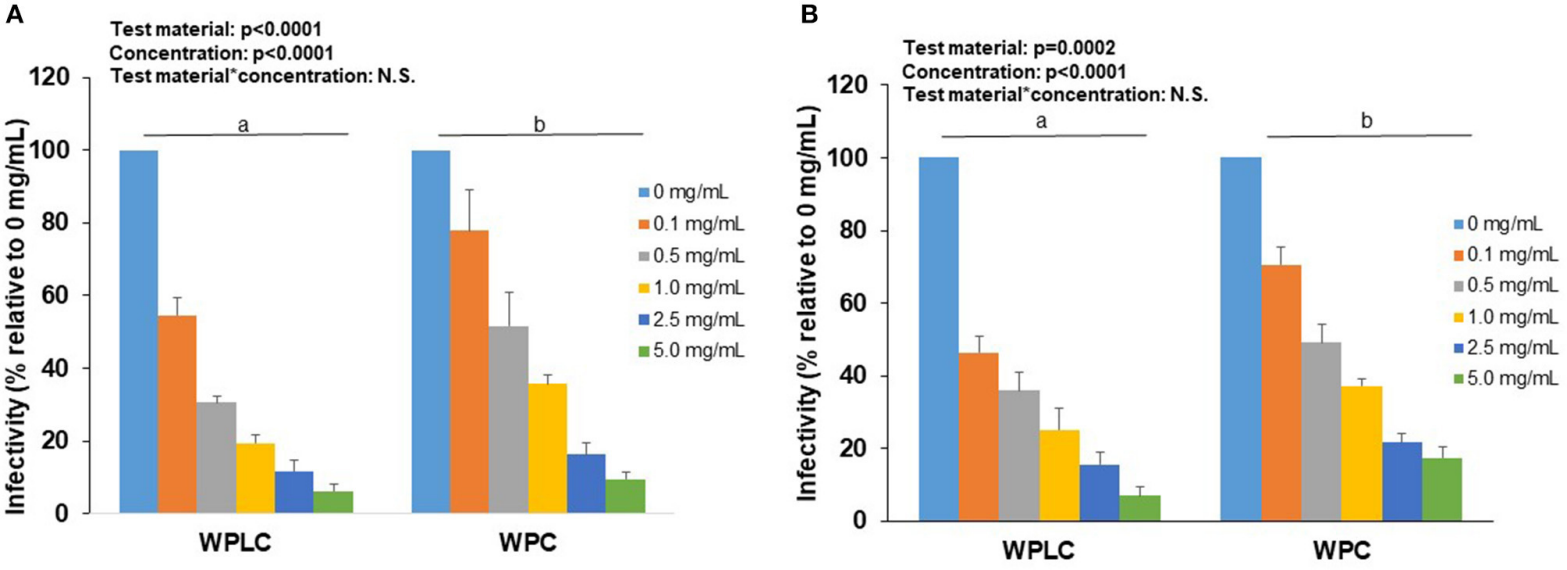
Concentration-dependent reduction of porcine RV OSU **(A)** or human RV Wa **(B)** strains infectivity in Caco-2 cells by WPLC and WPC. Data are represented as means ± SEM of 2–3 separate experiments. Infectivity was calculated as % of FFU at each concentration relative to FFU counted in cells infected with RV alone (0 mg/mL). Statistical analysis assessed test material, concentration and test material*concentration effects. Unlike letters represent significant differences (*p* < 0.05) between WPLC and WPC. RV, rotavirus; WPC, whey protein concentrate; WPLC, whey protein lipid concentrate.

**Figure 4:**
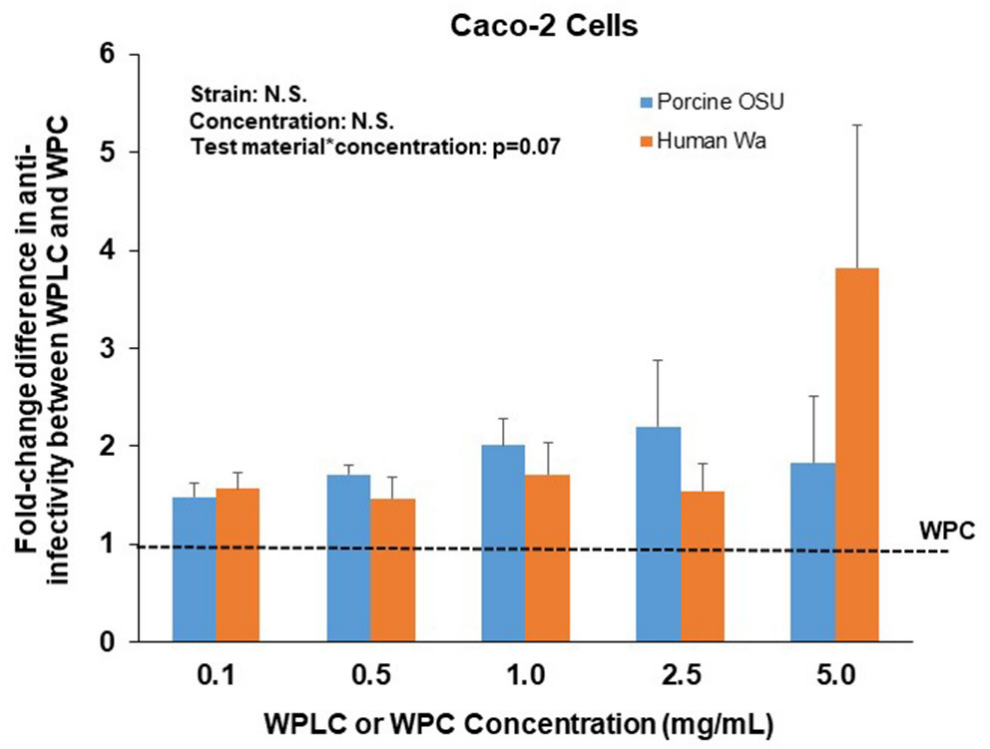
Fold-change difference in anti-RV activity of WPLC and WPC in Caco-2 cells infected with porcine RV OSU or human RV Wa. Data are represented as means ± SEM of three separate experiments. Fold-change represents the ratio between WPC and WPLC efficacy to reduce % infectivity at each concentration tested. At similar concentrations, WPLC efficacy over WPC to reduce infectivity of both strains OSU and Wa was equal in Caco-2 cells. RV, rotavirus; WPC, whey protein concentrate; WPLC, whey protein lipid concentrate.

Similar to the findings in MA104 cells, both WPLC and WPC caused a significant concentration-dependent reduction in % infectivity in Caco-2 cells (Table 2). Infectivity of porcine OSU decreased from 55–6.3% to 78–9.5% with increasing concentration of WPLC and WPC, respectively. In Caco-2 cells, human Wa RV infectivity ranged from 46.3 to 7.0% when incubated with WPLC and 71 to 18% with WPC. Both test material and concentration had significant independent effects, but statistical analysis indicated no interaction between those variables (Figure 3). There was, however, a trend toward significance in the interaction between test material and concentration when we compared the effectiveness (in fold-change) of WPLC vs. WPC (Figure 4). In Caco-2 cells, the fold-change reduction in porcine OSU and human WA infectivity was similar among the WPLC concentrations, and varied between 1.5 and 3.8. Again, WPLC was more effective than WPC in reducing RV infectivity under all conditions tested (i.e., fold-change difference >1 in all cases).

**Table 2:** Porcine RV OSU and Human RV Wa Infectivity (%) in Caco-2 Cells modulated by co-incubation with either WPLC or WPC.

**RV Strain**	**Test material**	**Concentration (mg/mL)**					
		**0**	**0.1**	**0.5**	**1.0**	**2.5**	**5.0**
Porcine OSU	WPLC	100^a^	54.7 ± 4.8^b^	30.6 ± 1.7^c^	19.4 ± 2.5^cd*^	11.7 ± 3.2^de^	6.3 ± 2.0^e^
	WPC	100^a*^	77.8 ± 11.3^a^	51.6 ± 9.4^bc*^	35.7 ± 2.6^c^	16.4 ± 3.0^d^	9.5 ± 1.9^d^
Human Wa	WPLC	100^a^	46.3 ± 4.4^b^	35.9 ± 5.3^bc^	25.2 ± 6.1^cd^	15.7 ± 3.2^d^	7.0 ± 2.5^d^
	WPC	100^a^	70.6 ± 4.7^b^^∧^	49.2 ± 4.9^bc^	37.3 ± 1.9^cd^^∧^	21.6 ± 2.6^d^	17.5 ± 3.1^d^

*Data is expressed as means ± SEM. Values are % inhibition of infectivity relative to 0 mg/mL*.

*Unlike letters indicate differences between the concentrations within each preparation at p < 0.05*.

* *Indicate statistical significance trend (0.5 > p < 0.1) between WPLC, 1.0 and 2.5 mg/mL; WPC, 0 and 0.1 mg/mL, and 0.5 and 1.0 mg/mL*.

∧ *Indicate statistical significance trend (0.5 > p < 0.1) between WPC, 0.1 and 0.5 mg/mL, and 1.0 and 2.5 mg/mL*.

**Figure 5:**
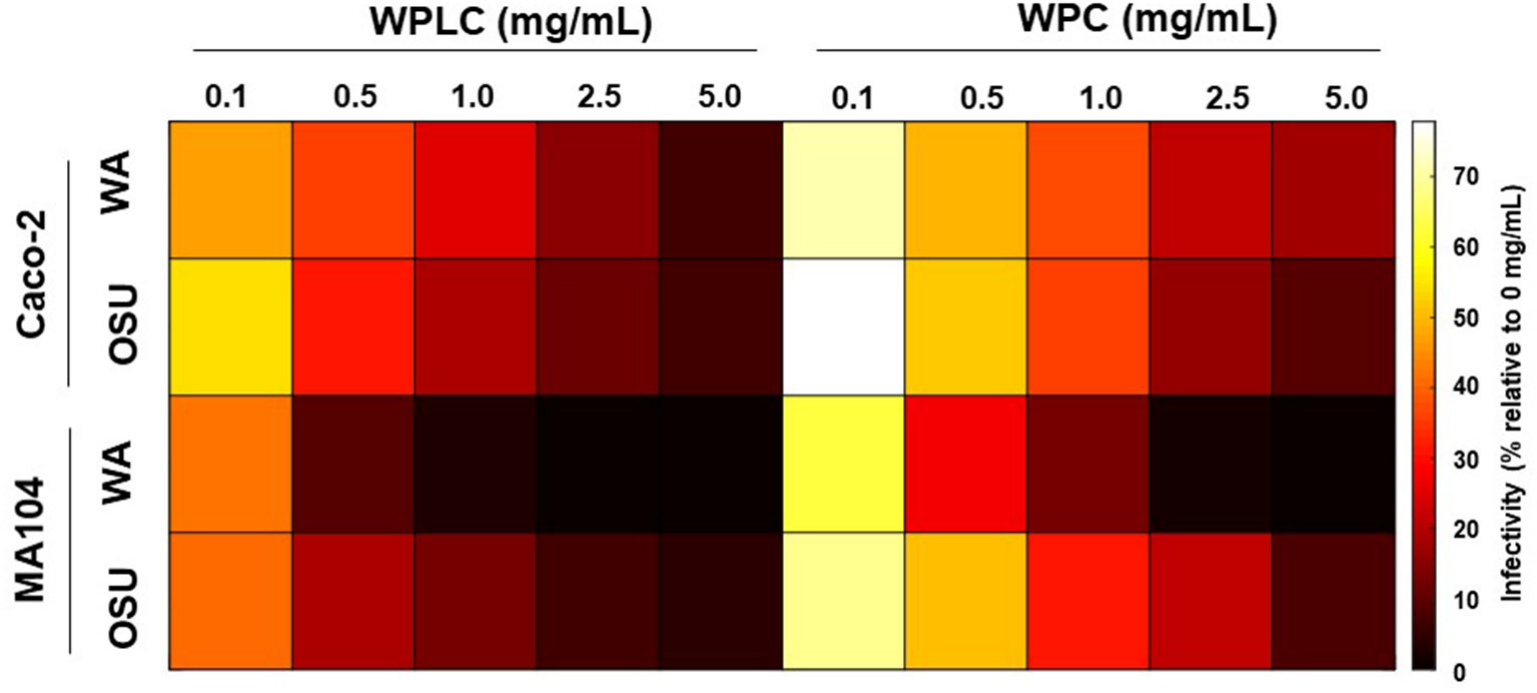
Heatmap representing WPLC and WPC efficacy in reducing porcine OSU and human Wa RV infectivity in MA104 and Caco-2 cells at concentrations ranging from 0.1 to 5.0 mg/mL (data shown in Tables 1, 2). Data are represented as % infectivity relative to control 0 mg/mL (scale on the right of the graph). Statistical analysis indicated independent and interactive effects (*p* < 0.01) of cell type, RV strain, test material, concentration, cell type*RV strains, cell type*concentration, RV strain*concentration, cell type* RV strain*test material*concentration. The interaction of cell type*test material was significant at trend level (*p* = 0.08), RV strain*test material was not statistically significant. RV, rotavirus; WPC, whey protein concentrate; WPLC, whey protein lipid concentrate.

To gain a more complete understanding of the differential responses to WPLC and WPC and the magnitude in their ability to reduce infectivity of two RV strains in two different cell types, a heatmap was constructed using the % infectivity data (Figure 5). As indicated in the results above, WPLC was more efficient in reducing infectivity than WPC at each of the concentrations tested, and was influenced by the cell type, but not the RV strain. Of the permutations tested, WPLC or WPC had their greatest impact in reducing infectivity when MA104 cells were infected with human Wa RV at concentrations >1.0 mg/mL with infectivity reduced to <1%. In Caco-2 cells, the differential response to WPLC or WPC were less affected by the RV strain than was observed in the MA-104 cells.

## Discussion

The MFGM is a natural component of bovine and human milk. Traditional methods of producing infant formulas have discarded the bovine milk fat in favor of vegetable oils (28). However, *in vitro*, preclinical, and clinical studies have demonstrated numerous potential health benefits of MFGM (9, 10). A systematic review and meta-analysis of MFGM supplementation in infants and children supported beneficial effects of MFGM on cognitive development and on reduction in otitis media incidence (29). Bolstered by these lines of evidence, bovine milk-derived MFGM preparations are available on a commercial scale from several sources and are added to some infant formulas currently on the market (13). Some caution has been raised based on the few numbers of clinical trials (28), but the overall body of evidence to substantiate beneficial health effects of MFGM is increasing.

In terms of gastrointestinal infections, evidence from *in vitro* studies support the ability of MFGM to inhibit pathogens, such as RV (14, 15, 30). However, evidence for the MFGM to inhibit infections in clinical studies is less conclusive and varied from a reduced longitudinal prevalence of diarrhea (31, 32) to no difference in diarrheal incidence between control and MFGM-containing experimental diets (33, 34). Thus, the overall conclusion of the systematic review and meta-analysis was that the effect of MFGM formula supplementation on diarrheal diseases, including RV, was conflicting and weak (29). The inconsistent findings from human studies may be explained by differences in MFGM formulations used in the supplementation studies. Different methods and approaches used to isolate MFGM from bovine milk can result in products that vary widely in overall protein, ash, and lipids composition and proportions of phospholipids types (35). The goal of this study was to test the anti-RV activity of a MFGM-enriched WPLC that is currently being added to infant formula. We compared the WPLC against a control WPC that was not enriched in MFGM and also tested batch variability. Lastly, we screened both WPLC and WPC against two strains of RV and MA104 and Caco-2 cell lines. The MA104 cell line is highly permissive to RV infection and is a well-characterized *in vitro* model to study RV mechanisms of action (36). The Caco-2 cell line has also been used for screening compounds for anti-RV activity (15, 37) and was selected herein to be more representative of intestinal epithelial cells vs. kidney epithelial cells (MA104).

Our data confirmed that for both WPLC and WPC, one of the batches obtained from the same manufacturers differed in their ability to reduce OSU strain RV infectivity in MA104 cells from the other two. It is reasonable to assume that the difference between the WPLC batches might have been related to variations in MFGM that reflect structural and compositional milk changes throughout lactation. Wilson et al. (38) demonstrated that the O-linked core of oligosaccharide profile of the bovine MFGM changed from colostrum to mature milk and suggested that the changes may be an adaptation to confer protection against pathogens during the early stages of life. The fat globule size could add to the variation, as MFGM protein and lipid composition alters with droplet size (39). Future research must consider the heterogeneous nature of commercial bovine MFGM and its potential impact on the formulation's functional properties. However, while there was a significant effect of batch in the current study, there was no interaction between batch and concentration, meaning that each WPLC batch inhibited RV in a concentration-dependent manner and did not differ at the same concentration tested among the three batches. Thus, we expect that different batches of WPLC from the same supplier material would have the potential to exert anti-RV activity.

We demonstrated that a commercially available ingredients material of WPLC (enriched with MFGM) effectively reduced RV infectivity in a concentration-dependent manner. However, we also observed that WPC alone reduced the ability of RV to infect cells *in vitro*, however WPLC was more effective with differences between WPLC and WPC most apparent at the lower concentrations of incubation. For example, WPLC inhibited ~60 % RV infectivity at the lowest concentration tested (0.1 mg/mL) and nearly 100% at the highest concentration (5 mg/mL) regardless of the cell line used and the RV strain. Our results agree with those reported by Kvistgaard et al. (15), in which human MFGM and WPLC decreased Rhesus monkey rotavirus (RRV) infectivity in MA104 cells. In that study, both human MFGM and WPLC reduced RRV infectivity by 91% infectivity at a protein concentration of 0.5 mg/mL compared to controls (no protein). At similar concentration, bovine MFGM reduced infectivity by 30% (15).

Unlike previous studies (15, 40), we compared the effectiveness of WPLC high in MFGM components as an RV inhibitor to WPC, an ingredient material that serves as a more adequate reference to compare bioactivity for MFGM-enriched ingredient materials given the whey-protein concentrate based nature of the materials. This comparison allows for identification of effects that are specifically elicited by the enrichment of MFGM components. WPC inhibited RV infectivity in both MA104 and Caco-2 cells, but it was less effective than WPLC at lower concentrations. For example, WPLC was 1.5- to 4.5-fold more efficacious in inhibiting RV infectivity, depending on the cell type. Bovine WPC contains various bioactive components with anti-infective properties, including lactoferrin, osteopontin, α-lactalbumin, and lactadherin, albeit in much lower concentrations than those found in human milk (41, 42). The data suggest the increased potency of WPLC over WPC is most likely due to the enrichment of the formulation with MFGM lipids and proteins. Previous work in our laboratory demonstrated that five non-polar lipid protein-free components isolated from buttermilk and cheese whey contributed to the anti-RV activity associated with the bovine MFGM (14). Furthermore, Parrón et al. (40) showed that buttermilk, butter serum, and MFGM neutralized bovine WC3 RV strain infectivity in MA104 cells. However, the percent neutralization decreased upon cream washing of the three test ingredients. As indicated by the authors, the study's caveat was that the cream washing procedure resulted in an unavoidable general loss of bound and loosely-bound proteins such as MUC1, lactadherin, butyrophylin, and the redox enzyme xanthine dehydrogenase/oxidase (40).

Others have shown that MFGM proteins prevent replication or the binding of viral particles to cells. Studies using a glycoprotein-enriched MFGM fraction containing MUC1, butyrophylin, and lactadherin demonstrated its ability to reduce replication of human Wa, KUN, and Mo RV strains (43) and RRV (44) strains. Interestingly, these two studies differed in their conclusion about which protein of the complex was responsible for its effect on RV. MUC1 was found to be the main component in the MFGM fraction to possess anti-viral activity against the human Wa, KUN, and Mo RV strains (45); whereas, Yolken et al. (44) attributed the reduction in RRV replication to lactadherin. A few years later, researchers were able to show that human but not bovine lactadherin inhibited human Wa RV infection via a protein-virus interaction (15) in Caco-2 cells. The same group found that bovine MUC1, but not lactadherin, had anti-viral activity against RRV in MA104 cells. Furthermore, a bovine macromolecular whey protein formulation similar to the WPLC used in our study reduced infectivity of both human Wa RV and RRV in Caco-2 and MA104 cells, respectively (15). The weak impact of individual MFGM compounds on RV may be linked to structural changes that may occur during isolation processes. Protein deglycosylation, heat treatment, and chemical hydrolysis of glycoprotein-bound sialic acid have all been shown to decrease anti-viral activity against RV (40, 44). Manufacturing processes of MFGM ingredient materials can impact structure and thereby bioactivity.

The mechanisms by which RV attaches to intestinal cells is complex and varies among RV strains reviewed in detail by Desselberger (45). For sialic acid (SA) -sensitive strains, such as the porcine OSU, the virus binds to cell receptors that contain SA in terminal or sub-terminal positions. In addition, SA-sensitive strains only infect efficiently through the apical surface of epithelial cells. Human RV strains, including the Wa strain studied in this study, are considered SA-resistant strains that bind cell receptors that contain SA in internal positions of glycolipids, and can enter the cell from both apical and basolateral sides (36). Human RV strains also bind to histo-blood group antigens, nonsialylated glycoconjugates, in a strain-specific manner (45). In our study, we incubated the human Wa or porcine OSU virus with WPLC or WPC prior to cell infection, suggesting that the reduction in RV infectivity resulted from the binding of WPLC or WPC components to the RV rather than the cell surface receptors. MFGM gangliosides are enriched with SA–containing compounds, such as *N*-glycolylneuraminic acid (Neu5Gc) and *N*-acetylneuraminic acid (Neu5Ac), which were previously reported to be present in the source of WPLC used in the current study at concentrations of 0.13 and 2.52%, respectively (46).

A key finding was that although WPLC reduced the infectivity of both RV strains in both cell lines, some differences were observed, with the greatest reduction observed in human Wa infectivity in MA104 cells, where 0.5 mg/ml resulted in nearly 90% inhibition of infectivity. While the human Wa strain RV likely is a better model for the types of RV strains that typically infect humans, the MA104 cells could be considered a less physiologically relevant model, since they do not express the complex morphological and functional characteristics of the human intestine. Caco-2 cells, although colonic epithelial cells, have the ability to differentiate into cells that resemble small intestinal epithelial cells. Interestingly, in this study the efficacy of WPLC to reduce the infectivity of the OSU and Wa RV strains in Caco-2 cells at highest concentrations was lower as compared to MA104 cells, which may reflect the complexity of the WPLC components that inhibit both SA-dependent and –independent strains of RV in a differentiated intestinal cell line.

## Conclusions

Innovation in infant formula composition may close the gap between the infection rates observed in breast-fed and formula-fed infants. Bovine-derived MFGM is currently being added to formula and have shown to provide some benefits to infants (13). We provided *in vitro* evidence that MFGM can efficiently reduce RV infectivity using different RV strains in two cell culture models. More studies are needed in preclinical animal models of RV infection (47) to demonstrate that these effects are also observed *in vivo* at the same concentrations present in human milk or MFGM-supplemented infant formula and to discern mechanisms of action. We also highlighted that the variability in the composition of MFGM formulation should be considered in the preparation of formula to maximize the efficacy of MFGM as bioactive component.

## Data Availability Statement

The datasets presented in this article are openly available. Requests to access the datasets should be directed to sdonovan@illinois.edu.

## Ethics Statement

The animal study was reviewed and approved by University of Illinois Urbana-Champaign Institutional Animal Care and Use Committee.

## Author Contributions

SD and GG: concept and design. MM: conducted experiments, collected data, data analyses, and manuscript writing. All the authors have approved the final version of the manuscript.

## Conflict of Interest

GG is an employee of Reckitt Benckiser/Mead Johnson Nutrition. The remaining authors declare that the research was conducted in the absence of any commercial or financial relationships that could be construed as a potential conflict of interest. The authors declare that this study received funding from Mead Johnson Nutrition/Reckitt Benckiser. The funder had the following involvement in the study: review of study design and review of the final manuscript prior to publication.

## Publisher's Note

All claims expressed in this article are solely those of the authors and do not necessarily represent those of their affiliated organizations, or those of the publisher, the editors and the reviewers. Any product that may be evaluated in this article, or claim that may be made by its manufacturer, is not guaranteed or endorsed by the publisher.
