# Supplementary material for: Whey Protein Lipid Concentrate High in Milk Fat Globule Membrane Components Inhibit Porcine and Human Rotavirus in vitro
Source: Front Pediatr. 2021 Sep 1;9:731005. doi: 10.3389/fped.2021.731005 (PMC8442734; doi:10.3389/fped.2021.731005)
Supplement: Supplementary file 1 [file Table_1.DOCX]

**Supplemental Table 1**. Protein content of Whey Protein Lipid Concentrate High in Milk Fat Globule Membrane (WPLC) and Whey Protein Concentrate (WPC) preparations corresponding to the doses used in the study,

| **Formulation** | **Protein Content**  **% wt/wt** | **Concentration (mg/mL)** | | | | |
| --- | --- | --- | --- | --- | --- | --- |
|  |  | **0.1** | **0.5** | **1.0** | **2.5** | **5.0** |
| WPLC-1 | 72.4 | 3.62 | 18.1 | 36.2 | 90.5 | 181.0 |
| WPLC-2 | 72.5 | 3.63 | 18.1 | 36.25 | 90.6 | 181.3 |
| WPLC-3 | 74.1 | 3.71 | 18.5 | 37.05 | 92.6 | 185.3 |
| WPC-1 | 79.0 | 3.95 | 19.8 | 39.5 | 98.8 | 197.5 |
| WPC-2 | 77.0 | 3.85 | 19.3 | 38.5 | 96.3 | 192.5 |
| WPC-3 | 79.0 | 3.95 | 19.8 | 39.5 | 98.8 | 197.5 |
