# Supplementary material for: Whey Protein Lipid Concentrate High in Milk Fat Globule Membrane Components Inhibit Porcine and Human Rotavirus in vitro
Source: Front Pediatr. 2021 Sep 1;9:731005. doi: 10.3389/fped.2021.731005 (PMC8442734; doi:10.3389/fped.2021.731005)
Supplement: Supplementary file 2 [file Data_Sheet_1.PDF]

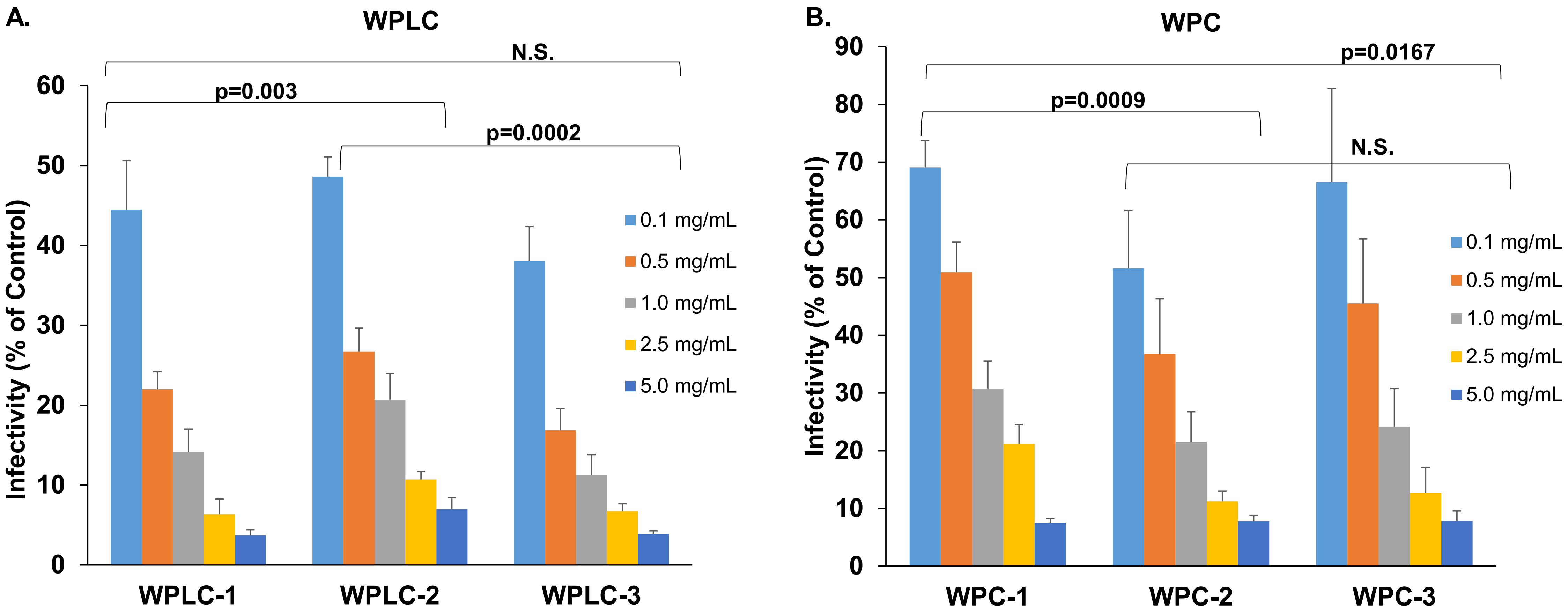


**Figure S1**. Variability in % infectivity by 3 batches each of WPLC (A) and WPC(B) formulations tested in MA104 cells infected with porcine OSU RV. Each bar represents the mean ± SEM of separate 3 experiments. Infectivity was calculated as % of FFU at each concentration relative to FFU counted in cells infected with RV alone (0 mg/mL). Statistical analysis assessed batch, concentration and batch*concentration effects. Batch interaction was not statistically significant. Despite of significant batch effect, there was no significant difference at same concentration tested among the 3 batches. Abbreviations: RV, rotavirus; WPC, whey protein concentrate; WPLC, whey protein lipid concentrate.
